# In-depth Temporal Transcriptome Profiling of Monkeypox and Host Cells using Nanopore Sequencing

**DOI:** 10.1038/s41597-023-02149-4

**Published:** 2023-05-09

**Authors:** Balázs Kakuk, Ákos Dörmő, Zsolt Csabai, Gábor Kemenesi, Jiří Holoubek, Daniel Růžek, István Prazsák, Virág Éva Dani, Béla Dénes, Gábor Torma, Ferenc Jakab, Gábor E. Tóth, Fanni V. Földes, Brigitta Zana, Zsófia Lanszki, Ákos Harangozó, Ádám Fülöp, Gábor Gulyás, Máté Mizik, András Attila Kiss, Dóra Tombácz, Zsolt Boldogkői

**Affiliations:** 1 Department of Medical Biology, Albert Szent-Györgyi Medical School, University of Szeged, Somogyi u. 4., 6720 Szeged, Hungary; 2 National Laboratory of Virology, Szentágothai Research Centre, University of Pécs, Pécs, Hungary; 3 Institute of Biology, Faculty of Sciences, University of Pécs, Pécs, Hungary; 4 Veterinary Research Institute, Hudcova 70, CZ-62100 Brno, Czech Republic; 5 Institute of Parasitology, Biology Centre of the Czech Academy of Sciences, Branisovska 31, CZ-37005 Ceske Budejovice, Czech Republic; 6 Department of Experimental Biology, Faculty of Science, Masaryk University, Kamenice, 753/5, Brno, CZ-62500 Czech Republic; 7 Department of Microbiology and Infectious Diseases, University of Veterinary Medicine Budapest, 1143 Budapest, Hungária krt. 23-25, Hungary

**Keywords:** Transcriptomics, Virus-host interactions, Pox virus

## Abstract

The recent human Monkeypox outbreak underlined the importance of studying basic biology of orthopoxviruses. However, the transcriptome of its causative agent has not been investigated before neither with short-, nor with long-read sequencing approaches. This Oxford Nanopore long-read RNA-Sequencing dataset fills this gap. It will enable the in-depth characterization of the transcriptomic architecture of the monkeypox virus, and may even make possible to annotate novel host transcripts. Moreover, our direct cDNA and native RNA sequencing reads will allow the estimation of gene expression changes of both the virus and the host cells during the infection. Overall, our study will lead to a deeper understanding of the alterations caused by the viral infection on a transcriptome level.

## Background & Summary

Monkeypox virus (MPXV) belongs to the *Poxviridae* family, which contains many viruses that infect various animal taxa including invertebrates, reptiles, and mammals. MPXV is the member of the human pathogenic *Orthopoxvirus* genus, which also includes the cowpox virus, the vaccinia virus (VACV) and the highly dangerous variola virus, the causative agent of smallpox^1,2^. Smallpox infections caused millions of deaths throughout the history until a global vaccination program has successfully eradicated the virus from the human population^3^. Infections of MPXV, have also been reported, although with lower mortality and milder morbidity^3^.

Monkeypox is a zoonotic pathogen, endemic to West and Central Africa and with the exception of some rare cases, human MPXV infections were localized only here during the last decades. However, due to a recent outbreak, a growing number of cases were reported from countries where the disease is not endemic^4,5^. The genomic monitoring of the 2022 MPXV outbreak revealed that the circulating MPXV strain is related to the less pathogenic West African clade of MPXVs but forms a highly divergent novel clade with an elevated mutation rate^6–8^. Consequently, the Public Health Emergency of International Concern (PHEIC) highlighted the epidemic potential of the virus outside its endemic region as well.

The orthopoxviruses are one of the largest of all animal viruses. Their virion is brick-shaped, membrane-coated and approximately 200–300 nm in diameter. Orthopoxviruses possess a large, linear double-stranded DNA genome, around 200 kbp in length^9^. In contrast to most other mammalian DNA viruses, which replicate in the nucleus (such as herpesviruses and adenoviruses), poxviruses remain in the cytoplasm. Viral DNA replication and the transcription of MPXV genes take place within compartments called “viral factories”, independently of the host cell^10^. This extraordinary feature draws attention to the means through MPXV regulates the gene expression of its host cell.

The transcriptional effect of MPXV infection on different cell types has been characterized before using micro-array-based techniques^11–13^. Rubins and colleagues used a high-resolution poxvirus-human microarray covering 24 h of infection and classified all MPXV genes for the first time according to their temporal expression^14^. They also compared the expression profile of MPXV to VACV and found that only the minority of transcripts are species-specific^14^. And though recent studies have re-evaluated these data using comparative pathway analyses, the detailed transcriptomic characteristics of MPXV-infected cells remains undescribed^15^. Thus, while micro-array-based techniques reveal useful insights, they are unable to resolve many aspects of the transcriptome, including the detection of the plethora of different transcript isoforms, which have been detected in closely related viruses, for example in VACV^16^.

RNA-sequencing has become the most widely applied method in transcriptome research. Short-read sequencing (SRS) techniques generate sufficient depth of sequencing and have a high accuracy, but transcriptome annotations may remain incomplete because of the fragmented nature of the sequenced cDNAs^17–19^. This is especially true in the case of viruses, which have gene-dense genomic regions where transcripts substantially overlap each other. Additionally, SRS has a severe limitation in distinguishing the different transcript isoforms^20^. Long-read sequencing methods (LRS), including Pacific Biosciences and Oxford Nanopore Technologies (ONT) offer an alternative for transcriptome sequencing that enables the recovery of full-length RNA molecules, which is invaluable for a precise transcriptome annotation^21^. Although these methods generate fewer reads and have higher error rates, compared to SRS, with sufficient read-depth, the assembly of complete transcriptomes of well-annotated genomes, like that of MPXV becomes possible^22–25^. Moreover, with the MinION platform it is possible to sequence native RNA molecules directly (dRNA-seq). This way the false products arising from either the reverse-transcription or PCR steps during the library preparation can be avoided. A drawback of dRNA sequencing technique however, is its inefficiency to precisely annotate the 5′ termini of mRNAs^26^. However, this problem can be overcome via the combined usage of dRNA-seq and 5′-end sensitive PCR-free direct cDNA sequencing methods (dcDNA)^22,27–29^. Furthermore, direct cDNA-seq can be used to accurately quantify gene expression, as it is not affected by biases introduced in the RT-PCR of traditional PCR-cDNA-sequencing^30^.

As of now, only a few transcriptomes have been analyzed by next generation sequencing (NGS) methods. This includes the VACV, a model for orthopoxviruses and a close relative of MPXV^31–35^. LRS methods have been used to redefine the highly intricate structure of VACV transcriptome^36^, moreover the dynamic gene expression changes were analyzed in detail during the time course of the infection^16,37,38^. However, to our best knowledge there is a lack of RNA sequencing datasets on the MPXV transcriptome. Hence, our goal in this work is to present an LRS dataset that will enable an accurate transcriptome annotation of MPXV.

In this study, the transcriptomes of the MPXV along with its host cell were sequenced using an Oxford Nanopore Technologies (ONT) MinION long-read sequencing device. Two sequencing approaches were utilized in this study: a dcDNA-seq of 6 different time-points (1-, 2-, 4-, 6-, 12- and 24-hours post infection) from the virus-infected cells, each with 3 biological replicates, and a dRNA-seq library from a mixture of the time-point samples.

This dataset can be used for the analysis of temporal transcriptomes of MPXV and the infected cells. Since even short-read transcriptomic data are completely missing of MPXV, our long-read RNA-seq dataset should serve as a gap-filler and will enable the in-depth characterization of its transcriptome. The transcriptomic landscape of human MPXV presented here will contribute to our better understanding of the virus and can ultimately aid the development of effective treatments in the future.

## Methods

**Fig. 1:**
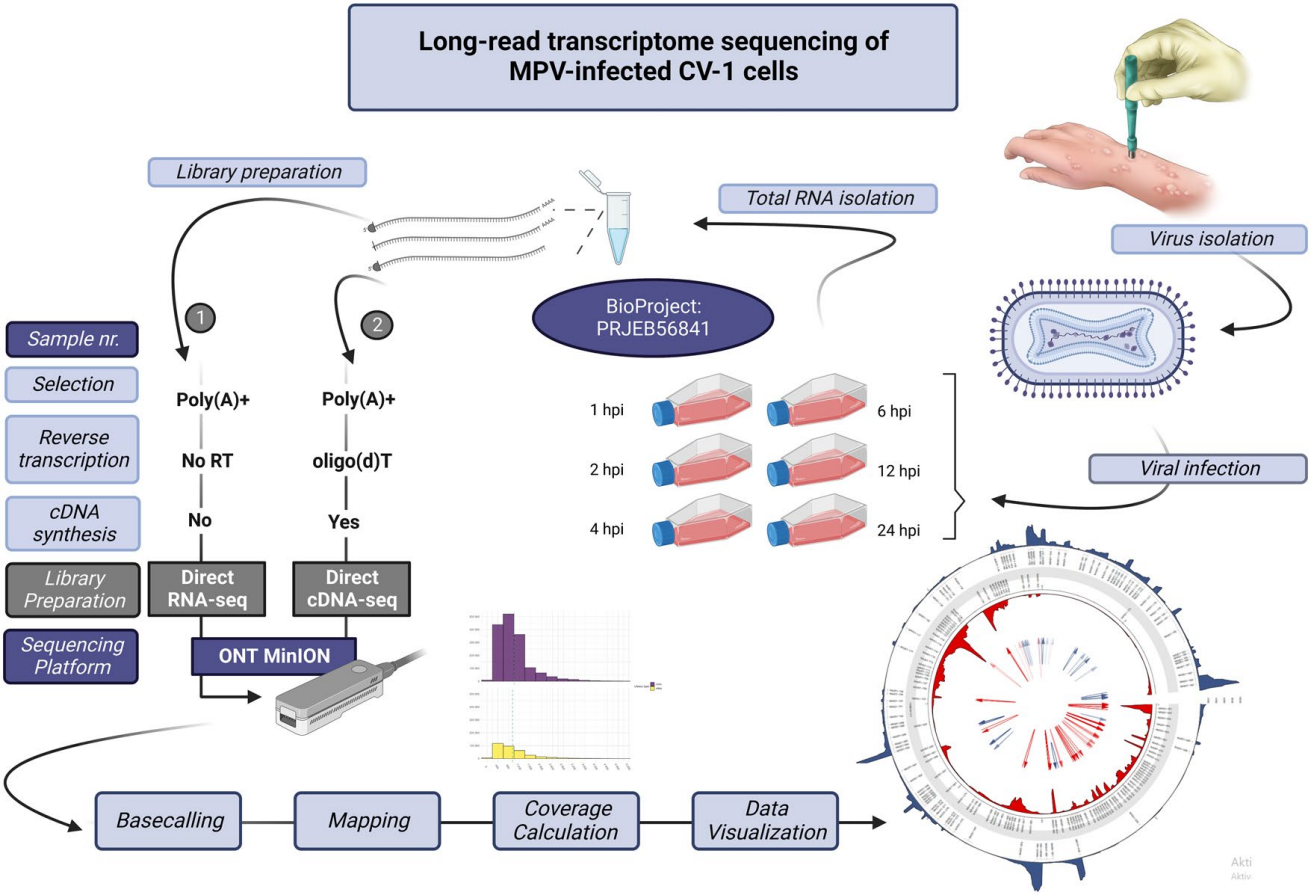
General overview of the study. Briefly, MPXV was isolated from a skin lesion and then was used to infect CV-1 cells. After the designated infection times, total RNA was isolated and sequenced using direct cDNA sequencing protocol on ONT’s MinION platform. The experiment was carried out in triplicates. A mixed time-point sample was also prepared and used for direct RNA sequencing. The reads were basecalled and then mapped to the viral and host genomes. From the alignments viral coverages were calculated and visualized. The figure was created with Biorender (BioRender.com).

Figure 1 shows the detailed workflow of the study.

### Cells

CV-1 (CCL-70, African green monkey, kidney) cell line was used which was obtained from American Type Culture Collection (ATCC). For the experiment 75 cm^2^ tissue culture flasks (CELLSTAR®; Greiner Bio-One GmbH, Frickenhausen, Germany) were plated with 2 × 10^5^ cells in Minimum Essential Medium Eagle culture medium (MEM) with 10% fetal bovine serum (FBS). The CV-1 cells were cultivated until ~80% (~1.2 × 10^6^) confluency at 37 °C in humified 5% CO_2_ atmosphere. Before the infection, the monolayer was washed with 1 X PBS (Thermo Fisher Scientific, Waltham, MA, USA).

### Collection, detection, isolation and propagation of the virus

The MPXV (MPXV_NRL 4279/2022) was isolated from skin lesions and kindly provided by Dr. Jirincova (The National Institute of Public Health, Prague, Czech Republic). All procedures with infectious materials were performed under BSL-4 conditions at the National Laboratory of Virology, University of Pécs. The virus was passaged once on CV-1 cells to reach a sufficient amount of infective particles. The same batch of working stock was used during the experiment. The viral titer of the working stock was determined with plaque assay on CV-1 cells. Non-infected control cultures were inoculated with MEM and treated the same way as the infected ones. For the infection, 2 ml MPXV with 5 plaque-forming units (pfu)/cell (MOI = 5) was used, which was diluted with MEM to reach the sufficient concentration. Cells were incubated with monkeypox inoculum at 37 °C for 1 hour while were shaken gently in every ten minutes. The virus inoculum was removed, then the cell monolayer was washed once with 1 x PBS. For the flasks 10 mL MEM medium was added which was supplemented with 2% FBS, 2 mM L-glutamine and 1% penicillin and streptomycin solution. The cells were incubated at 37 °C for 1, 2, 4, 6, 12 and 24 hours in a humidified 5% CO_2_ atmosphere. Each time, the experiment was done in triplicate and subjected to direct cDNA sequencing. Prior to direct RNA sequencing extra flask was used to sample the following time points: 2-, 6-, 12- and 24-hours post-infection. Direct RNA sequencing was carried out without replicates. After the incubation, the supernatant was removed, and the cells were washed with PBS. The dry flasks were stored at −80 °C until further processes. The cells were washed and scraped down into lysis buffer and transferred to 1,5 mL Eppendorf Tubes® (Thermo Fisher Scientific, Inc.).

### Isolation of total RNA

Total RNA was purified from the MPXV-infected and from mock-infected CV-1 cells at various time points after infection from 1 to 24 hours. For this, the NucleoSpin RNA Kit (Macherey-Nagel) was used, following the manufacturer’s recommendations. Briefly, cells were collected by centrifugation (1000 × g), then 350 µl RA1 lysis buffer (part of the NucleoSpin RNA Kit) and 3.5 µl β-Mercapthoethanol (Sigma Aldrich) were added to the samples and then, mixtures were centrifuged at 11,000 × g for 1 min in NucleoSpin Filter tubes. Filters were discarded, and the lysate was washed using 70% EtOH (350 µl) on NucleoSpin RNA Column with centrifugation at 11,000 × g for 30 sec. Membrane Desalting Buffer (350 µl, from the NucleoSpin RNA Kit) was then added to desalt the membrane, which was finally dried with centrifugation (11,000 × g). Residual DNA was removed using rDNase enzyme [rDNase:rDNase reaction buffer (1:9 ratio, NucleoSpin Kit)]. The enzymatic reaction was carried out at room temperature (RT) for 15 min. The NucleoSpin Kit’s RAW2 Buffer (200 µl) was used on the NucleoSpin Filter, which inactivated the enzyme. After a short centrifugation (11,000 × g, 30 min) the Filter was placed in a new Eppendorf tube. The next washing step was carried out with RAW3 Buffer (600 µl, from the NucleoSpin RNA Kit) and centrifugation (11,000 × g, 30 min). This step was repeated with 250 µl RAW3 Buffer. The purified total RNA samples were eluted from the Filter in 60 µl nuclease-free water (NucleoSpin RNA Kit) and they were stored at −80 °C (Table 1).

**Table 1:** RNA quantities. Obtained yield of total RNAs and polyA-selected RNAs (ng/µl).

dcDNA					dRNA		
Sample Name	Biological Replicate	Hours past infection	RNA yield (ng/µl)		Hours past infection	RNA yield (ng/µl)	
			total RNA	polyA-selected		total RNA	polyA-selected
1 h/A	A	1	598	14.7	1	830	12.4
1 h/B	B	1	1060	11.4	1		
1 h/C	C	1	856	13.4	1		
2 h/A	A	2	672	14.2			
2 h/B	B	2	698	13.1			
2 h/C	C	2	492	14.9			
4 h/A	A	4	1080	11.7			
4 h/B	B	4	1040	11.6			
4 h/C	C	4	932	11.2			
6 h/A	A	6	1000	12.3	6	994	12.2
6 h/B	B	6	942	12.9	6		
6 h/C	C	6	988	12.5	6		
12 h/A	A	12	760	13.1	12	738	14.4
12 h/B	B	12	624	14.0	12		
12 h/C	C	12	1060	11.4	12		
24 h/A	A	24	1020	11.8	24	906	12.4
24 h/B	B	24	1000	13.5	24		
24 h/C	C	24	788	14.3	24		
Mock/A	A	0	738	12.8			
Mock/B	B	0	880	11.8			
Mock/C	C	0	784	12.0			

Right panel: RNA samples used for dcDNA sequencing; left panel: RNAs from which the dRNA-seq sample was mixed.

### Poly(A) selection

Polyadenylated RNA was enriched using the Lexogen’s Poly(A) RNA Selection Kit V1.5. This method is based on oligo(dT) beads, which hybridize RNAs with polyadenylated 3′ ends, but RNAs without poly(A) stretches (e.g. rRNAs) do not captured by the beads and therefore, they will be washed out. The applied protocol is as follows: the beads (from of the Lexogen Kit) were resuspended and 4 µl for each RNA samples was used. Beads were collected in a magnet, and the supernatant was discarded. RNAs were resuspended in Bead Wash Buffer (75 μl, Lexogen Kit) and then were placed on the magnet, and supernatant was discarded. This washing step was repeated. Beads were resuspended in RNA Hybridization Buffer (20 μl, Lexogen Kit). Ten μg from the total RNA samples were diluted to 20 µl in nuclease-free water (UltraPure™, Invitrogen) and then they were denatured at 60 °C for 1 min. Denatured RNA samples were mixed with 20 µl beads. The mixtures were incubated in a shaker incubator with 1250 rpm agitation at 25 °C for 20 min. Next, the samples were placed in a magnetic rack. Supernatant was discarded, the tubes were removed from the magnet, the collected samples were resuspended in 100 µl Bead Wash Buffer (Lexogen Kit), and finally, they were incubated for 5 min at 25 °C with 1250 rpm agitation. Supernatant was discarded and this washing step was repeated once. Beads were resuspended in 12 µl nuclease-free water, then kept at 70 °C for 1 min. After this incubation step, tubes were placed on a magnetic rack and supernatant, containing the polyadenylated fraction of RNA samples were placed to new DNA LoBind (Eppendorf) tubes (Table 1). Samples were stored at −80 °C.

### Direct cDNA sequencing

Direct (d)cDNA libraries were generated with the aim of analyzing the dynamic pattern of MPXV transcripts and the effect of viral infection on the host cell gene expression profile. RNA samples from different time points (1, 2, 4, 6, 12 and 24 h p.i., and from the mock, three biological replicates from each) were used individually for library preparation. The ONT’s Direct cDNA Sequencing Kit (SQK-DCS109, ONT) was applied according to the manufacturer’s recommendations. Briefly, first-strand cDNAs were synthesized from the polyA(+) RNA samples using the Maxima H Minus Reverse Transcriptase enzyme (Thermo Fisher Scientific) and the SSP and VN primers (supplied in the ONT kit). The potential RNA contamination was eliminated by applying RNase Cocktail Enzyme Mix (Thermo Fisher Scientific).

The second cDNA strands were generated with LongAmp Taq Master Mix (New England Biolabs). The ends of the double-stranded cDNAs were repaired with NEBNext End repair/dA-tailing Module (New England Biolabs) and then the adapters were ligated using the NEB Blunt/TA Ligase Master Mix (New England Biolabs). The Native Barcoding (12) Kit (ONT) was used for multiplex sequencing. The samples (200 fmol/flow cell) were loaded onto MinION R9.4 SpotON Flow Cells (ONT, Table 2).

**Table 2:** Sequencing barcodes and amount of libraries (in µl) used for sequencing.

Sample Name	Flow cell #	Barcode #	Barcode sequence	Amount of library used for sequencing (µl)
1 h/A	1	BC01	AAGAAAGTTGTCGGTGTCTTTGTG	7.20
1 h/B		BC02	TCGATTCCGTTTGTAGTCGTCTGT	7.74
1 h/C		BC03	GAGTCTTGTGTCCCAGTTACCAGG	7.44
2 h/A		BC04	TTCGGATTCTATCGTGTTTCCCTA	8.48
2 h/B		BC05	CTTGTCCAGGGTTTGTGTAACCTT	6.26
2 h/C		BC06	TTCTCGCAAAGGCAGAAAGTAGTC	5.53
4 h/A	2	BC07	GTGTTACCGTGGGAATGAATCCTT	8.87
4 h/B		BC08	TTCAGGGAACAAACCAAGTTACGT	8.81
4 h/C		BC09	AACTAGGCACAGCGAGTCTTGGTT	7.09
6 h/A		BC10	AAGCGTTGAAACCTTTGTCCTCTC	7.70
6 h/B		BC11	GTTTCATCTATCGGAGGGAATGGA	10.71
6 h/C		BC12	CAGGTAGAAAGAAGCAGAATCGGA	7.65
12 h/A	3	BC13	AGAACGACTTCCATACTCGTGTGA	5.15
12 h/B		BC14	AACGAGTCTCTTGGGACCCATAGA	6.87
12 h/C		BC15	AGGTCTACCTCGCTAACACCACTG	5.60
24 h/A		BC16	CGTCAACTGACAGTGGTTCGTACT	5.36
24 h/B		BC17	ACCCTCCAGGAAAGTACCTCTGAT	6.32
24 h/C		BC18	CCAAACCCAACAACCTAGATAGGC	4.56
Mock/1	4	BC19	GTTCCTCGTGCAGTGTCAAGAGAT	15.39
Mock/2		BC20	TTGCGTCCTGTTACGAGAACTCAT	15.85
Mock/3		BC21	GAGCCTCTCATTGTCCGTTCTCTA	13.66

Two-hundred fmol dcDNA library mixture was loaded onto each of the Flow Cells (33.34 fmol/sample from viral infected samples and 66.67 fmol from the mock-infected libraries).

### Direct RNA sequencing

Direct RNA sequencing (SQK-RNA002; Version: DRS_9080_v2_revO_14Aug2019, Last update: 10/06/2021) was used to sequence the native RNA strands to avoid any potential bias from reverse transcription or PCR. Fifty ng (in 9 μl) from a mixture of polyA(+) RNAs from various time points (2, 6, 12 and 24 h p.i.) was used for library preparation. As a first step, 1 μl RT Adapter (110 nM; ONT Kit) was ligated to the RNA sample using 3 μl NEBNext Quick Ligation Reaction Buffer (New England BioLabs), 0.5 μl RNA CS (ONT Kit), and 1.5 μl T4 DNA Ligase (2 M U/ml New England BioLabs) at RT for 10 min. The first cDNA strand was generated using SuperScript III Reverse Transcriptase (Life Technologies), as recommended by the Direct RNA sequencing (DRS) manual (ONT). The reaction was carried out at 50 °C for 50 min and it was followed by the inactivation step at 70 °C for 10 min. Next, the sequencing adapters (ONT’s DRS kit) were ligated to the cDNA at RT for 10 min using the T4 DNA ligase enzyme and NEBNext Quick Ligation Reaction Buffer. The dRNA library was sequenced on an R9.4 SpotON Flow Cell.

RNAClean XP beads and AMPure XP beads (both from Beckman Coulter) were used after each of the enzymatic reactions for washing the dRNA-seq and dcDNA-seq libraries, respectively.

### Bioinformatics

The generated sequencing reads were basecalled with the Guppy software (available at ONT’s community site https://community.nanoporetech.com/), with the following parameters:–flowcell FLO-MIN106–kit SQK-DCS109–barcode_kits EXP-NBD114–min_qscore 8–recursive–calib_detect. Based on a quality threshold of 8, the basecalled reads were separated into a ‘pass’ and a ‘fail’ group – the subsequent analyses were carried out on the passed reads. The .fastq files containing the passed reads for the respective samples were merged.

The resulting sequences were then mapped to a combined reference, containing the host genome (GenBank assembly accession: GCF_015252025.1^39^) and the viral genome (GenBank assembly accession: GCA_023516015.3^40^, GenBank nucleotide accession: (ON563414.3^41^), using minimap2^42^. The reference genomes were downloaded from NCBI GenBank. The mapping parameters were the following: minimap2 -ax splice -Y -C5–cs–MD -un -G 10000. The generated .bam files were uploaded to the European Bioinformatics Institute’s European Nucleotide Archive (EBI ENA) under the following BioProject ID: **PRJEB56841**^43^ and to the Sequence Read Archive (SRA) under accession **ERP141806**^44^. Supplementary Table S1 contains the ENA accession IDs and read files uploaded to ENA.

**Fig. 2:**
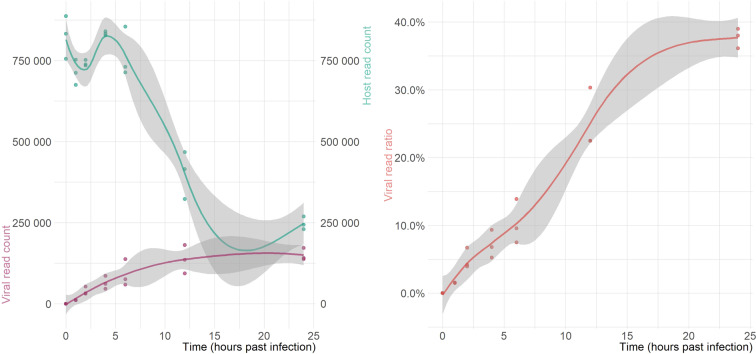
Sequencing read counts and viral read ratios in the dcDNA samples. In the left panel purple dots represent the number of viral reads, while green dots represent the number of host reads in each biological replicate. In the right panel, the dots represent the ratio of viral reads to the total read count per sample. The colored lines in both panels represent the result of a smoothing function, while the grey lines represent 95% confidence intervals. A clear decrease in the host reads and an increase in the viral read ratio shows the progress of the viral infection.

**Fig. 3:**
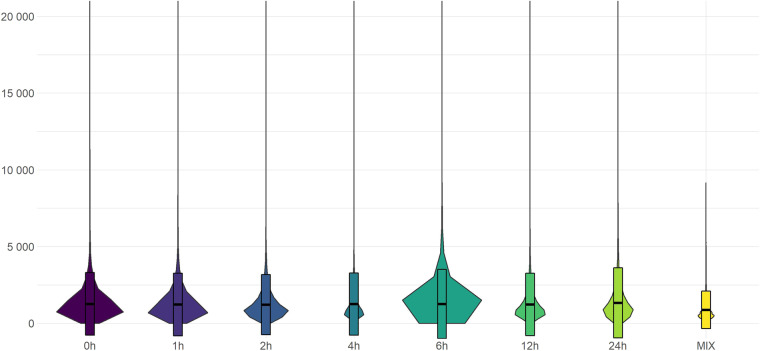
Violin plot iluustration of the read length distributions in the cDNA and the dRNA sequencing libraries. The hinges of the added boxes correspond to the first and third quartiles of the data, and the bold line indicating the median values.

**Fig. 4:**
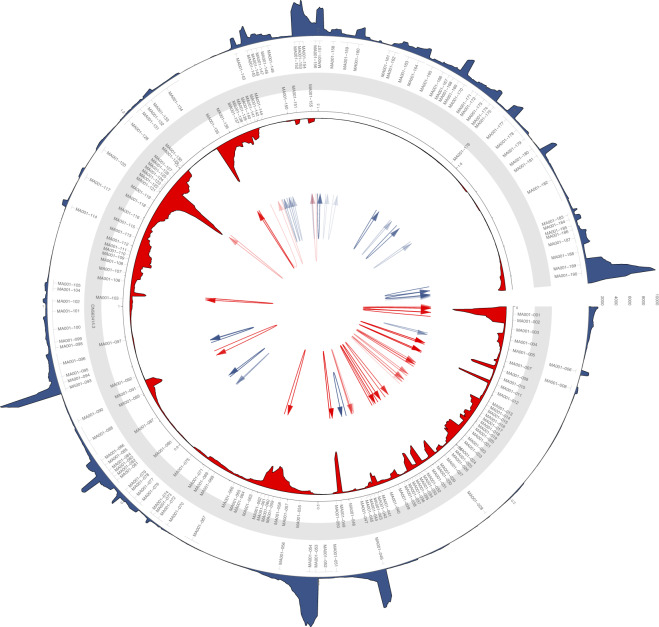
Coverage of the viral genome in the dRNA sequencing library. The mean coverage on the monkeypox genome was calculated in a 100-nt window. The links in the center of the circle represent transcripts, as in the connections between the 5′- and 3′-ends of the reads. These potential ‘transcripts’ were filtered to read count threshold of 10. The transparency of the links is correlated with the abundance of the ‘transcripts’.

**Fig. 5:**
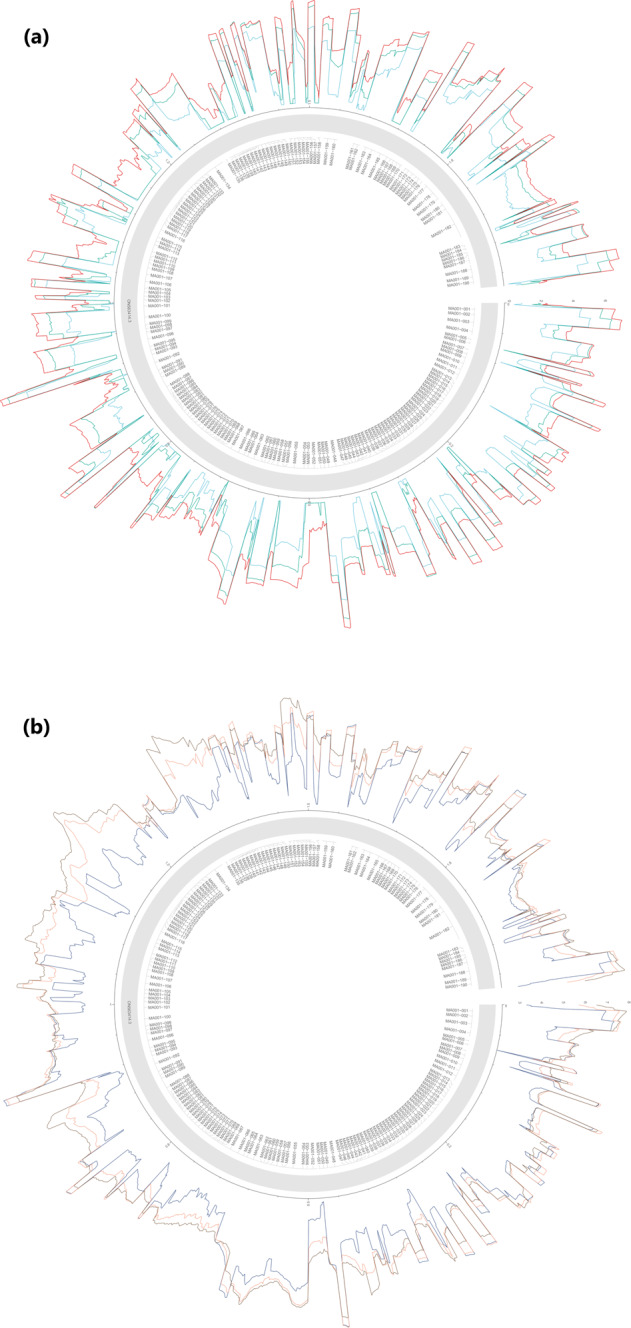
Log10 transformed coverage of the viral genome in the dcDNA sequencing library. (**a**) Coverage in 1-, 2- and 4-hours post-infection; (**b**) coverage in 6-, 12- and 24-hours post-infection.

The subsequent analyses were carried out within the R environment – all scripts are available in our GitHub repository https://github.com/Balays/MPOX_ONT_RNASeq^45^. The workflow implements functions from the tidyverse^46^ collection of R packages. The complete workflow can be re-run to produce all the analysis results, including generation of figures and tables. The first step in the MPOX-wf is to import the .bam files into the R workspace using Rsamtools^47^. Raw alignment counts were calculated using *idxstats*. Then reads with secondary alignments were filtered out, as these are putatively chimeric RNAs. Viral and host read counts, according to the mapping results (Fig. 2) and read lengths (Fig. 3 and Supplementary Figure S1) were visualized with the ggplot2 package^48^. Next, per-base coverage values and their statistics across the whole genome and also in 100 nt windows were calculated. Supplementary Table S2 contains the mean, median and standard deviation of the coverage of each time-point across the whole genome, while Supplementary Table S3 contains the more detailed (per-window) coverage statistics. The coverages were used for generating Supplementary Figure S2 and Supplementary Figure S3. The gene arrows for the genome annotation were generated using gggenes (https://github.com/wilkox/gggenes). The mean coverage on monkeypox genome in the dRNA sample and in the dcDNA samples (after *log10* normalization) was visualized using the circlize package^49^ (Fig. 4 and Fig. 5, respectively). The links in the center of the circle represent transcripts, as in the connections of the 5′- and 3′-ends of the reads. These putative transcripts were filtered to a read count threshold of 10. The transparency of the links is correlated with the abundance of the transcripts.

## Data Records

Data (bam files containing the alignment and the sequence and its quality information as well) were uploaded to the EBI’s European Nucleotide Archive (ENA), under the following BioProject: **PRJEB56841**^43^ and the files are located at NCBI SRA under accession **ERP141806**^44^. Metadata of the uploaded files are available in the Supplementary Table S1. All data can be used without restrictions. In the case of dcDNA samples, from each time point, three biological replicates were generated; these were named according to the following scheme: 1h_A, 1h_B, 1h_C, 2h_A, …; where the ‘h’ stand for hours past infection (hpi).

The 21 dcDNA sequencing yielded a substantial amount of 15,062,290 reads that passed guppy’s QC filtering threshold of 8 (Table 3) and could be mapped onto either the host or to the viral reference genome (Fig. 2, left panel). The distribution of the lengths of these reads are shown in Fig. 3 and of the viral reads in Supplementary Figure S1. The mean of the read lengths did not change significantly, most of the reads were in the 800–1000 nt bin.

**Table 3:** Sequencing summary.

sample	Hours past infection	Library Type	Host read count	Viral read count (nochimaera)	Viral read ratio
mock_A	0 h	cDNA	755484	0	0.00000
mock_B	0 h	cDNA	832329	0	0.00000
mock_C	0 h	cDNA	886821	0	0.00000
1 h_A	1 h	cDNA	675122	10713	0.01562
1 h_B	1h	cDNA	752425	11501	0.01506
1h_C	1h	cDNA	711744	10798	0.01494
2 h_A	2 h	cDNA	738043	32320	0.04195
2 h_B	2h	cDNA	734619	52820	0.06708
2h_C	2h	cDNA	752317	30664	0.03916
4 h_A	4 h	cDNA	827627	45877	0.05252
4 h_B	4h	cDNA	840378	61162	0.06784
4h_C	4h	cDNA	833005	85903	0.09348
6 h_A	6 h	cDNA	730476	59224	0.07500
6 h_B	6h	cDNA	854951	137766	0.13878
6h_C	6h	cDNA	713161	75437	0.09566
12 h_A	12 h	cDNA	415440	180882	0.30333
12 h_B	12h	cDNA	467509	135560	0.22478
12h_C	12h	cDNA	322945	93748	0.22498
24 h_A	24 h	cDNA	230171	141024	0.37992
24 h_B	24h	cDNA	269179	171988	0.38985
24h_C	24h	cDNA	243493	137664	0.36117
dRNA	MIX	dRNA	576622	318802	0.35603

The ‘Host read count’ column shows the number of reads mapped onto the Vero genome; while the ‘Viral read count’ column shows the number of reads that were mapped to the ON563414.3 genome^41^, and that did not have secondary alignments (as these are potentially chimeric reads). The ‘Viral read ratio’ column corresponds to the ratio of these non-chimeric viral reads and the sum of the host and viral reads.

The ratio of viral reads showed a steady increase from around 1.52% ± 0.036% in the 1 hpi samples to 37.70% ± 1.45% in the 24 hpi samples (Fig. 2, right panel). The median coverage across the whole viral genome also increased: from 11 to 571 (Fig. 5). The total read count peaked at 4- and 6-hours post-infection and decreased afterwards. We observed a remarkable cytopathic effect after 12 hours, which reached a significant level on the cell monolayer and disrupted the coherence of cells. Most cells were perished at or after this time point. This is supported by the significant decrease in the host read counts and the increase in the viral read ratio.

The dRNA sequencing yielded 576,622 host and 318,802 reads of viral origin, corresponding to a 35.6% of viral read ratio and a mean coverage of 244 across the viral genome (Fig. 4). The two sequencing libraries compromise a total of 1,793,855 and 13,408,375 good quality viral and host reads, respectively.

## Technical Validation

### RNA

Qubit RNA BR and HS Assay Kits (Invitrogen) were used to measure the amount of total RNA and polyA-selected RNA samples, respectively. The final concentrations of the RNA samples were determined by Qubit 4.0.

### cDNA

**Fig. 6:**
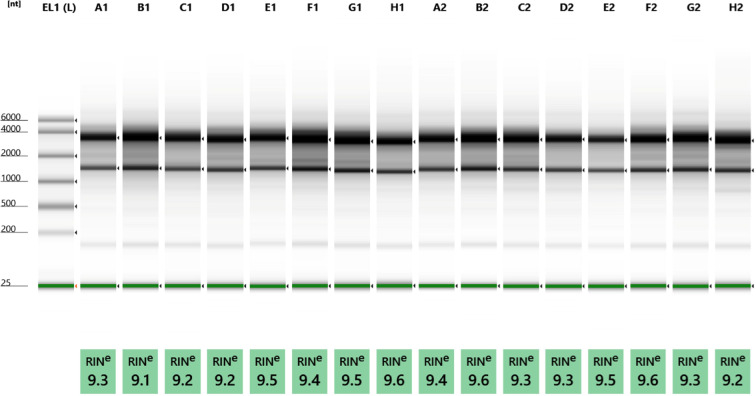
Quality of total RNA samples. The quality of the RNAs were assessed by using a TapeStation 4150 System and RNA ScreenTape (both from Agilent Technologies). TapeStation gel image shows that intact, high-quality RNAs (RIN > 9) were isolated from the cells and used for Nanopore sequencing. The image shows the following samples: EL1(L): marker; A1: 1 h (replicate); B1: 1 h (replicate C); C1: 2 h (replicate A); D1: 2 h (replicate B); E1: 4 h (replicate A); F1: 4 h (replicate B); G1: 6 h (replicate A); H1: 6 h (replicate B); A2: 12 h (replicate A); B2: 12 h (replicate C); C2: 24 h (replicate A); D2: 24 h (replicate B); E2: 2 h (used for dRNA-seq); F2: 6 h (used for dRNA-seq); G2: 12 h (for dRNA-seq); H2: 24 h (for dRNA-seq).

The amount of the cDNA samples and the ready cDNA libraries were measured using Qubit 4.0 fluorometer and Qubit dsDNA HS Assay Kit (Invitrogen). The quality of RNA was detected with the Agilent 4150 TapeStation System. RNA samples with RIN values ≥ 9.0 were used for sequencing (Fig. 6).

Three biological replicates were used for each of the infection time points. To analyze the effect of MPXV infection on the transcriptome profile of the host cells, mock-infected CV-1 cells were also harvested and sequenced.

## Usage Notes

Our dataset can be used to annotate novel viral transcripts and transcript isoforms, but possibly from the host as well. There are several bioinformatic tools that can be used to achieve this, including: TALON^50^; LIQA^51^; LoRTIA (https://github.com/zsolt-balazs/LoRTIA); EPI2ME’s transcriptomes workflow (https://github.com/epi2me-labs/wf-transcriptomes) or SQUANTI3 (https://github.com/ConesaLab/SQANTI3^52^). Transcript annotation can be carried out from both types of sequencing data (dcDNA and dRNA), however as dRNA-seq yields less artificial or false products, it is suggested to use these reads for validating the dcDNA-seq derived transcripts^30^. Although it is possible that some rare transcripts that are expressed in a subset of the time-points exclusively (e.g., some immediate early isoforms) could not be captured in the dRNA sequencing library. After identification, the novel transcripts should be annotated to ORFs, their coding capacity be estimated, their TSS and TES sites be analyzed and accordingly their isoform categories be assessed (long or short TSS, alternative termination, etc.).

The gene-wise and/or transcript-wise gene counts from the cDNA-seq data can be subjected to differential gene expression (DGE) or differential transcript expression (DTE), respectively. Furthermore, differential transcript usage analyses (DTU) can be carried out as well, for example with RATS^53^. The https://github.com/nanoporetech/pipeline-transcriptome-de pipeline, based loosely on the workflow presented in^54^, carries out these analyses from the annotated transcriptome, while EPI2ME’s transcriptomes workflow (https://github.com/epi2me-labs/wf-transcriptomes) carries out the transcript annotation and the above analyses in succession. The DGE, DTE and DTU analyses can be carried out both on the viral and on the host data and they can be based upon several comparisons, for example mock vs each time-point. In addition, the longitudinal expression data from cDNA-seq can be subjected to a time-series analysis as well^55^.

Besides focusing on individual genes or transcripts, gene-set enrichment analysis (GSEA) or pathway enrichment analyses can also be carried out to identify biological pathways that are affected by the viral infection in the host cells, for example with pathfindR^56^.

A combined workflow would be: 1.) detect transcripts using both sequencing approaches, but 2.) use the dRNA reads for validation, 3.) annotate them and carry out the transcript isoform analyses, 4.) quantify these validated transcripts in the cDNA data to estimate transcript counts, and finally 4.) carry out the above mentioned DGE, DTE, DTU and biological pathway analyses. Taken together, the almost 1.5 million viral and almost 13 million host reads enable the in-depth and temporal characterization of the Monkeypox transcriptome and the effect of the viral infection on the host gene expression.

## Supplementary information

Supplementary Figures

Supplementary Table 1

Supplementary Table 2

Supplementary Table 3

## Data Availability

The complete workflow, from mapping to the generation of figures is available at the GitHub repository (https://github.com/Balays/MPOX_ONT_RNASeq).
