# Supplementary material for: In-depth Temporal Transcriptome Profiling of Monkeypox and Host Cells using Nanopore Sequencing
Source: Sci Data. 2023 May 9;10:262. doi: 10.1038/s41597-023-02149-4 (PMC10170163; doi:10.1038/s41597-023-02149-4)

Figure legend

Supplementary Figure 1. Viral read length density distributions. A) Density plot for each sample with mean values shown as red dashed lines; B) Histogram for the combined cDNA and dRNA libraries; and C) Violin plot with added boxplots for each sample.

Supplementary Figure 2. Coverage of the viral genome in each sampling time points. The coverage was calculated for both DNA strands and three replicates were summed for each panel. The viral CDSs are shown in red (+ strand) and with blue (- strand) gene arrows.

Supplementary Figure 3. Log10 transformed coverage of the viral genome. Same as in Supplementary Figure 2, except the read coverages were log10 transformed.

Supplementary Figure 1

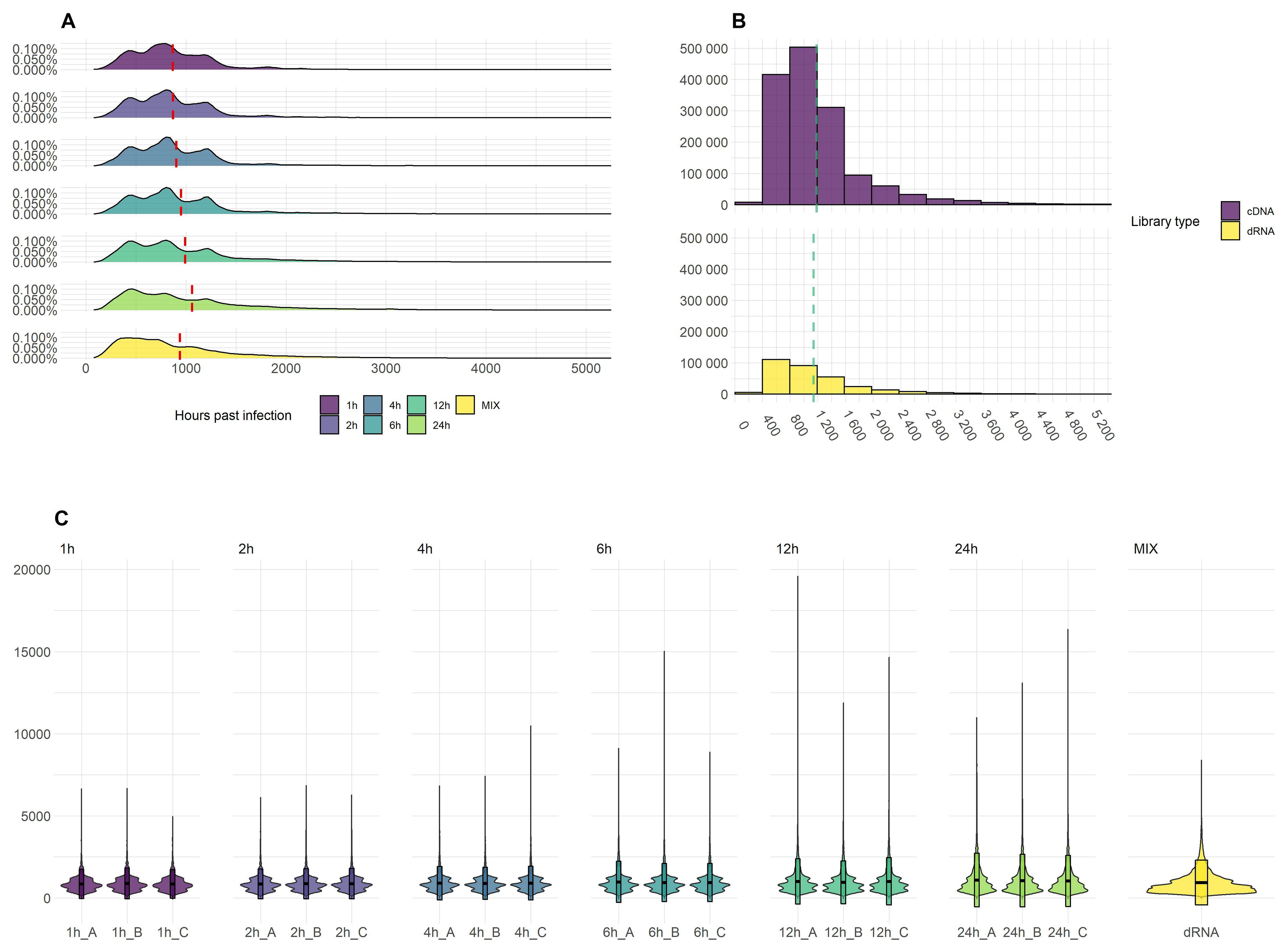

Supplementary Figure 2

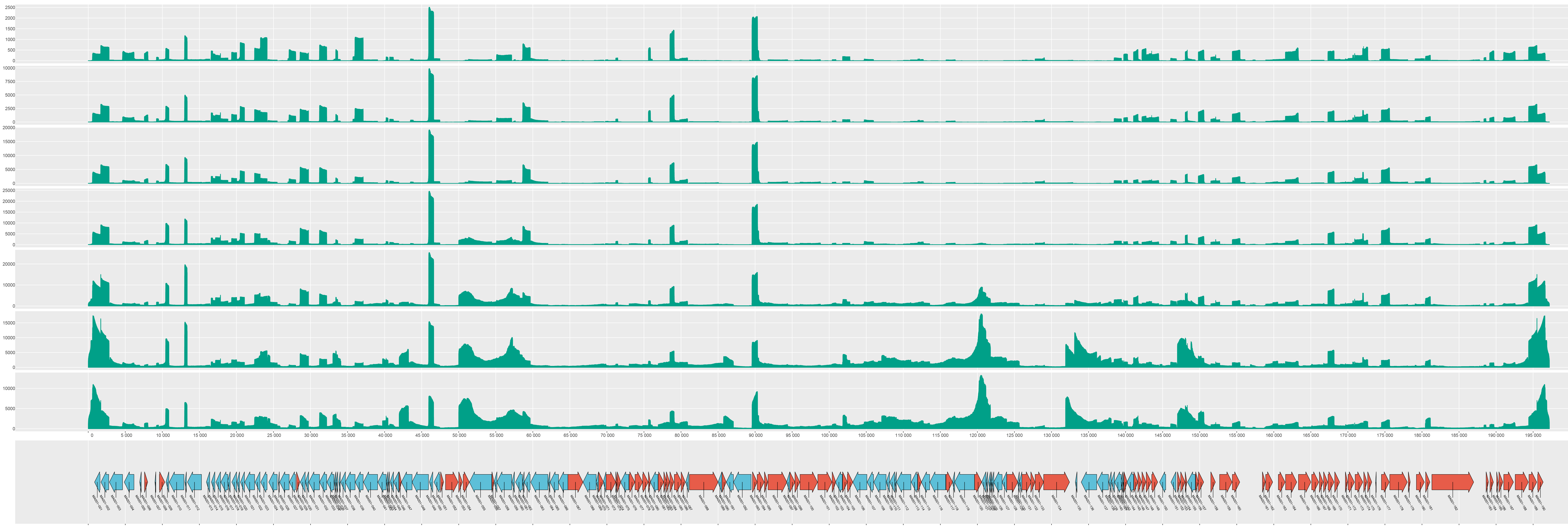

Supplementary Figure 3

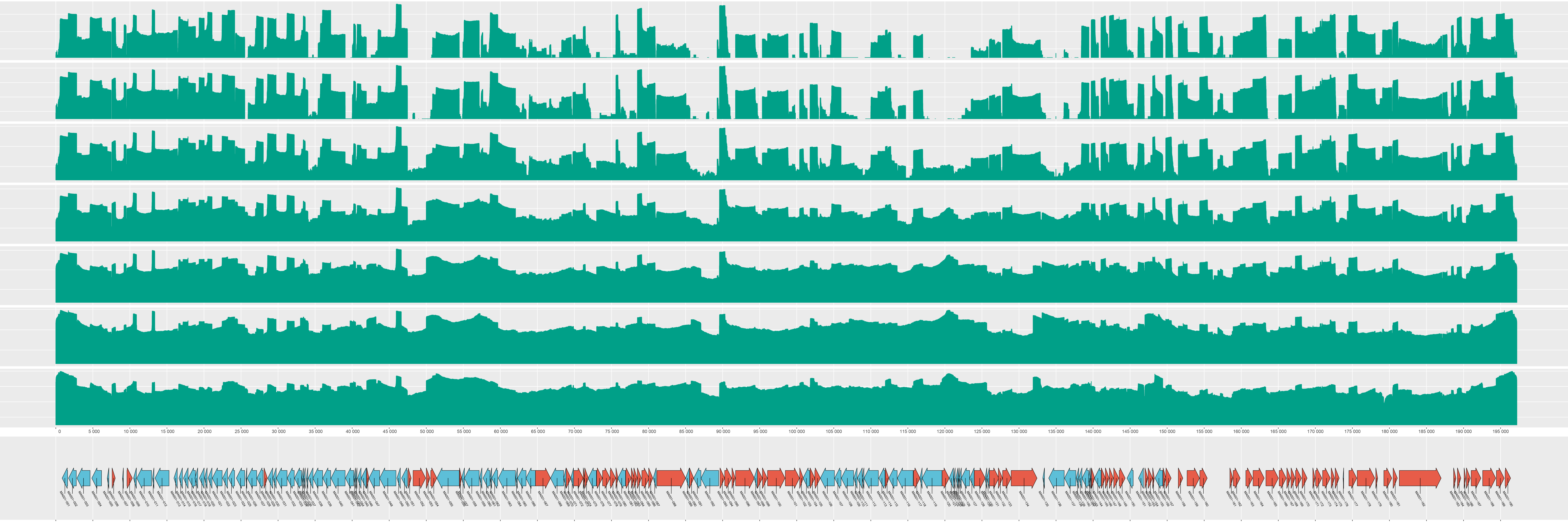

Supplement: Supplementary file 1 — Supplementary Figures [file 41597_2023_2149_MOESM1_ESM.pdf]
